# Artificial light at night and warming impact grazing rates and gonad index of the sea urchin *Centrostephanus rodgersii*

**DOI:** 10.1098/rspb.2024.0415

**Published:** 2024-04-17

**Authors:** Amelia Caley, Ezequiel M. Marzinelli, Maria Byrne, Mariana Mayer-Pinto

**Affiliations:** ^1^ Centre for Marine Science and Innovation; Evolution and Ecology Research Centre; School of Biological, Earth and Environmental Science, University of New South Wales, Sydney, New South Wales 2052, Australia; ^2^ School of Life and Environmental Sciences, The University of Sydney, Sydney, New South Wales, Australia

**Keywords:** herbivory, seaweed, multiple stressor, consumption, echinoderm

## Abstract

Artificial light at night (ALAN) is a growing threat to coastal habitats, and is likely to exacerbate the impacts of other stressors. Kelp forests are dominant habitats on temperate reefs but are declining due to ocean warming and overgrazing. We tested the independent and interactive effects of ALAN (dark versus ALAN) and warming (ambient versus warm) on grazing rates and gonad index of the sea urchin *Centrostephanus rodgersii.* Within these treatments, urchins were fed either ‘fresh’ kelp or ‘treated’ kelp. Treated kelp (*Ecklonia radiata*) was exposed to the same light and temperature combinations as urchins. We assessed photosynthetic yield, carbon and nitrogen content and C : N ratio of treated kelp to help identify potential drivers behind any effects on urchins. Grazing increased with warming and ALAN for urchins fed fresh kelp, and increased with warming for urchins fed treated kelp. Gonad index was higher in ALAN/ambient and dark/warm treatments compared to dark/ambient treatments for urchins fed fresh kelp. Kelp carbon content was higher in ALAN/ambient treatments than ALAN/warm treatments at one time point. This indicates ocean warming and ALAN may increase urchin grazing pressure on rocky reefs, an important finding for management strategies.

## 1. Introduction

Artificial light at night (ALAN) is increasingly disrupting natural light cycles, with profound consequences for ecosystems across the globe [1]. Over the last century, ALAN has been steadily increasing due to the rise of electrical lighting [2], posing an unprecedented threat to natural ecosystems. While artificial light provides many benefits to humans, it also disrupts natural light/dark cycles, causing changes in the behaviour [3,4], distribution [5], physiology and survival of species [6,7]. To date, much of the research into the impacts of ALAN has focused on terrestrial species [1,8]. However, ALAN is increasingly recognized as a key threat to marine ecosystems [8], with over 22% of coastlines impacted by ALAN globally [9]. This figure is predicted to increase due to increasing coastal population growth [10] and use of light-emitting diodes (LEDs) [11].

Most studies on the impacts of ALAN have focused on effects on individuals or populations [1,12]. However, ALAN is likely to not only impact multiple species within a particular community, but also to affect species interactions such as grazing and predation [13,14]. Therefore, ecosystem-level impacts of ALAN will vary depending on the species affected as well as on the magnitude and direction of these effects. For example, ALAN can simultaneously increase both biofilm biomass and gastropod grazing rates in intertidal ecosystems, resulting in no net change to biofilm biomass [14]. ALAN is also likely to co-occur with other stressors such as ocean warming, which may interact to alter the magnitude and direction of effects of ALAN. Manipulative experiments that test interactions between ALAN and other stressors and examine species interactions across trophic levels are needed to better understand the impacts of ALAN in terrestrial and marine systems.

Kelps are large habitat-forming seaweeds that underpin key ecosystem services, supporting high levels of biodiversity [15], fisheries productivity [16,17] and primary productivity [18]. Globally, major threats to kelp forests include ocean warming and heatwaves [19], as well as increased grazing by marine herbivores due to range shifts or predator release [20–22]. However, as kelp forests are a key feature of many coastal urbanized habitats (e.g. [23]) they are subject to many additional stressors besides warming, including ALAN [24]. Nevertheless, the impacts of ALAN on kelp have yet to be assessed [8]. Kelp photosynthesis and growth are controlled by light availability [25], so ALAN may have positive effects on kelp at the individual level. However, ALAN may also act like other stressors, such as warming, to negatively impact kelp, either directly (e.g. by reducing survival, biomass and photosynthesis [26–28]) or indirectly (by increasing the palatability of kelp to marine grazers by altering kelp nutritional content, microbial community and chemical defences [29,30]).

Sea urchins are key grazers in marine habitats, as they control the abundance of seaweeds and thereby exert a strong influence on ecosystem structure and biodiversity. When urchins reach high abundances, their grazing can cause a shift in dominant habitat from kelp forests to urchin barrens (e.g. [31]). These animals are also harvested for their gonads and support economically and culturally significant fisheries globally [32]. There is therefore a strong interest in factors that influence urchin grazing rates and gonad index. Warming can increase urchin metabolic rates, grazing rates and gonad index [27,33–35], but research into ALAN impacts on urchins is limited (but see [36] and [37]). Urchins can detect light via photoreceptors in their tube feet (e.g. [38]), and many species exhibit diel hiding/foraging behaviour, potentially to avoid predators [39–41]. This diel behaviour can vary between lunar cycles, demonstrating their high photosensitivity [42]. ALAN may alter foraging behaviour of urchins by disrupting these diel cues [36], or by changing metabolic rates of urchins due to increased stress [43]. This could alter the top-down control of urchins on kelp ecosystems. Day length is also an important cue for gonad development and spawning in urchins [33,44,45]. Therefore, ALAN may alter urchin gonad development, with possible impacts to their reproductive potential [46] and the marketability of urchin gonads [47]. Previous studies have found that ALAN reduces grazing rates and gonad index of *Heliocidaris crassispina* [37], reduces foraging behaviour of *Paracentrotus lividus*, and has no effects on *Arbacia lixula* [36]. This highlights the importance of assessing ALAN impacts on different species of urchins as well as conducting longer-term experiments, as urchins can display behavioural and physiological plasticity in response to stressors (e.g. [48]. Additionally, many studies examining herbivore-producer trophic relationships in marine systems expose either herbivores (e.g. [36,37]) or primary producers (e.g. [30,49]) to stressors individually, but not in combination, limiting the understanding of the mechanisms driving observed changes.

*Centrostephanus rodgersii* is a large diadematid urchin native to south-eastern mainland Australia, New Zealand and some offshore islands [50], and is an important consumer of *Ecklonia radiata*, the main habitat-forming kelp in southern Australia [51]. This sea urchin is also a target species for commercial fisheries in eastern Australia [52–54]. Since the 1970s, the range of *C. rodgersii* has expanded southwards from the Australian mainland to Tasmania [55], facilitated by changes in larval dispersal due to the strengthening poleward flow of the East Australian Current (EAC) [56] and the wide thermal tolerance of this species [57]. In high abundances, *C. rodgersii* naturally forms urchin barrens [58] and this range extension has resulted in the creation of new barrens habitat in Tasmania [21]. Warming can increase somatic growth and increase the kelp thinning capacity of *C. rodgersii* [27], however the effects of ALAN on *C. rodgersii* have not been studied. *C. rodgersii* may be particularly sensitive to ALAN as it is a nocturnal grazer [40,58] and as Diadematid sea urchins are highly sensitive to light [38,59]. Spawning in *C. rodgersii* coincides with short day lengths and low temperatures (austral winter [46]). Therefore, there may be interactive effects of ALAN and warming on *C. rodgersii* grazing and gonad index. With trends of increasing ocean warming and ALAN expected to continue, it is important to understand how these stressors will affect temperate Australian reefs through changes in key trophic interactions.

We experimentally tested the independent and interactive effects of ALAN (ALAN versus dark) and warming (warm versus ambient) on grazing rates and gonad index of *C. rodgersii*. We also tested if effects were consistent whether urchins were fed ‘fresh’ or ‘treated’ kelp’ (*Ecklonia radiata*) (i.e. urchins were fed kelp exposed to the same respective light and warming treatments as urchins). We also assessed effects of ALAN and warming on the photosynthetic yield, carbon and nitrogen content and C : N ratio of treated kelp, to identify potential drivers of effects on urchin gonad index and grazing. We predicted that grazing rates would be lower in ALAN treatments, potentially due to reduced time spent grazing at night, and higher in warming treatments due to increased metabolic rates, thus masking impacts on treatments where urchins were exposed to both ALAN and warming (i.e. zero net effect). We predicted that effects on grazing would be similar whether urchins were fed fresh kelp or kelp exposed to the same light and temperature treatments (treated kelp), and that effects of ALAN would lessen over time as urchins adjusted to treatments. We predicted that the gonad index of urchins would be higher in warm treatments compared to ambient treatments due to higher grazing rates, but that this effect would be smaller for urchins fed treated kelp due to negative effects of warming on kelp (e.g. altered nutritional content). Conversely, we predicted that gonad index would be lower in ALAN treatments due to reduced grazing rates, and that this effect would be greater for urchins fed treated kelp due to negative effects of ALAN on kelp (e.g. altered nutritional content). Finally, we hypothesized that ALAN would increase kelp photosynthetic yield, but warming would decrease photosynthetic yield; and that both warming and ALAN would reduce C : N ratio and C and N content of treated kelp.

## 2. Methods

### (a) Collection and acclimation

Fifty-six urchins were collected at 4 m depth from a rocky shore in Sydney Harbour, Australia (33°49'48.0″ S, 151°15'43.7″ E). This site contains *E. radiata* kelp beds and small urchin barrens and does not receive direct ALAN (personal observation and [60]). Urchins had a mean test diameter of 78.18 cm ± 1.47 s.e. (range: 55 mm–106 mm), which is representative of the most common sizes of *C. rodgersii* found in Sydney [61,62]. All urchins were collected on 6 June 2022 using SCUBA, placed in cool boxes full of seawater and transported by boat to aquaria facilities of the nearby Sydney Institute of Marine Science (SIMS). In the laboratory, urchins were randomly assigned to separate 45 l tanks (one urchin per tank) (electronic supplementary material, figure S1). Each tank contained a terracotta flowerpot to provide refuge for the urchins to avoid direct light exposure (electronic supplementary material, figure S1), as *C. rodgersii* typically shelter in crevices during the day.

Urchins were acclimatized for two weeks from collection to set a common baseline of feeding and to assess post-collection condition. During the acclimation period, tanks were lit for 10 h a day using cool white LEDs (150 cm cool white LED tubes 6500 K from MakeMyLED) with a light intensity of approximately 3000 lux (measured at the water's surface), and a 30 min sunrise and sunset to simulate natural light regimes at the time of the experiment (austral winter). Warm treatments were gradually heated to a target temperature of 2°C above ambient water temperatures (electronic supplementary material, figure S1) during the acclimation period, reflecting the projected sea temperature increase under the RCP 4.5 emission stabilization scenario [63]. Mean temperatures over the experiment are described below. In the final week of acclimation, overhead lighting was reduced to approximately 1500 lux in the day to better reflect daytime light levels at 4 m depth. Urchins were fed three times a week with *E. radiata* collected from a nearby rocky shore at Sydney Harbour, Chowder Bay (33°50'21.9″ S, 151°15'18.6″ E).

### (b) Light and temperature treatments

We manipulated ALAN and temperature across a total of 56 tanks. Urchins were randomly allocated to treatments, and two-factor ANOVAs confirmed urchin diameter did not differ among light and temperature treatments for fresh or treated kelp (electronic supplementary material, table S1, *p* > 0.17). After two weeks of acclimation, half of the urchin tanks (*n* = 28) were assigned to ‘dark’ treatments, which consisted of a 10 h light (1500 lux, measured at the surface of the water) cycle from 7 am–5 pm and 14 h dark (less than 0.1 lux) cycle with a 30 min sunrise and sunset to simulate the natural light regime at the time of the experiment. The remaining tanks (*n* = 28) were assigned to ‘ALAN’ treatments, with a light cycle of 10 h light during the day (1500 lux, as per control treatment) and 14 h of dim light during the night (50 lux, measured at the surface of the water). This light intensity is within the range used by other studies looking at the effects of ALAN on urchins (30 lux [36]; 300 lux [37]). While the lux used here is brighter than ALAN levels measured on some intertidal shores in Sydney Harbour [60], it is within relevant levels of light that can occur directly under infrastructure such as ports and marinas [13]. Importantly, the levels applied here are useful for understanding the mechanisms and potential long-term effects of ALAN [8]. Black vinyl was used to cover tanks to prevent spillover of light between treatments. Half of the tanks in each light treatment (i.e. dark and ALAN) had unmanipulated temperatures (ambient), while half were heated to a target temperature of 2°C above ambient temperatures (warm), totalling 14 tanks per light/warming treatment. Temperature was allowed to fluctuate naturally to mimic natural temperature variability (electronic supplementary material, figures S2 and S3). Mean temperature was 17.8 ± 0.002°C (range of 15.1°C–22.4°C) in ambient treatments, and 18.8 ± 0.002°C (range of 16.0°C–23.2°C) in warm treatments (electronic supplementary material, figure S2). Temperatures were measured every 30 min using HOBO loggers attached to tank airlines (electronic supplementary material, figures S2 and S3).

### (c) Kelp feeding treatments

To determine whether changes in grazing rates, if any, were due to changes in urchin behaviour and/or metabolism, kelp palatability or both, we had two additional kelp treatment categories, ‘fresh kelp’ and ‘treated kelp’. Urchins in the ‘fresh kelp’ treatment were fed with *E. radiata* collected twice a week from Chowder Bay and kept at ambient conditions at the aquaria tanks, while urchins in the ‘treated kelp’ treatment were fed *E. radiata* that had been exposed to the same respective light and temperature treatments as the urchin replicates (dark/ambient, dark/warm, ALAN/ambient, ALAN/warm). Most urchin replicates (*n* = 10) were fed with ‘fresh kelp’, whereas, due to limited space availability, only a subset of urchin replicates from each treatment (*n* = 4) were fed with ‘treated kelp’.

For the ‘treated kelp’ category, juvenile *E. radiata* of length approximately 15 cm [30] were collected on 16 June 2022 at 1–2 m depth from Chowder Bay, Sydney. Kelp was attached to plastic mesh using cable ties secured around the kelp stipe and placed in 60 l tanks, with approximately 30 individuals in each of the eight tanks. Kelp was acclimated for four days, during which warm treatments were gradually heated to 2°C above ambient using two 300 W Eheim Jager aquarium heaters per tank. Kelp treatments were randomly assigned to light and temperature treatments (dark/ambient, dark/warm, ALAN/ambient, ALAN/warm), with *n* = 2 tanks per treatment. The treated kelp was then fed to urchins in the respective treatment. In the kelp tanks, mean temperature was 17.3 ± 0.01°C (range of 15.1°C to 18.4°C) in ambient treatments and 18.8 ± 0.01°C (range of 16.8°C to 20.6°C) in warm treatments (electronic supplementary material, figure S3). Due to a decline in quantity and quality of kelp held in tanks over the experiment duration, kelp was collected again before weeks 5 and 7 of the experiment and acclimated to treatments for four days before being fed to urchins. These collections groups were each analysed separately as below (collection 1, 2 and 3).

### (d) Urchin condition and survival

Urchins were fed ad libitum with kelp for the experimental duration, and tanks were cleaned three times a week to remove debris. Due to the large number of tanks, sampling was staggered by one day for half the tanks, distributed randomly across treatments, for the entire experiment. Experimental treatments commenced when sampling started for each of the replicates (i.e. lights were turned on for ALAN treatments). Survival of urchins was 100% for the duration of the experiment (11 weeks, including the two-week acclimation period).

### (e) Urchin grazing rates

Urchin grazing rates (wet weight of kelp consumed in 24 h) were measured twice for each replicate tank in each measurement week (weeks 1, 2, 5, 7 and 9). In the first two weeks, sampling was done weekly to assess whether responses to light and warming happened rapidly to inform planning. After that, sampling was conducted fortnightly for the rest of the experiment. To quantify grazing rates, an approximately 10 g piece of kelp was blotted dry with paper towel, weighed using digital scales (0.1 g), then placed in each urchin treatment tank. After 24 h, the remaining kelp was collected, blotted dry and weighed to measure quantity consumed. Mass-corrected grazing rate was calculated for each replicate for each sample week as in Donham *et al*. [64] using the equation, Wi−Wf/Mi×t, where *Wi* is the initial kelp wet weight, *Wf* is the final kelp wet weight, *Mi* is the mass of the individual urchin, and *t* is time as days. This was done to account for differences in grazing rate due to urchin size [64]. The quantity of kelp (6–8 g) provided in week one was insufficient, as some urchins consumed all the kelp within 24 h. Thereafter, the kelp quantity was increased to 10 g day, to ensure there was kelp remaining in all replicates after 24 h. Urchins were not weighed prior to feeding trials to avoid disturbance, but they were weighed at the conclusion of the experiment to calculate the mass-corrected grazing rate (see details below).

### (f) Urchin weight, diameter and gonad index

At the end of the experiment, i.e. after nine weeks, the test diameter of each individual urchin was measured to 0.01 mm using digital callipers, and the whole wet weight of urchins was measured to 0.1 g using digital scales. Urchins were then dissected, and gonads were removed. The wet weight of gonads was measured to 0.1 g using digital scales. Gonad index was calculated using the following formula:

$$\mathrm{Gonad}\;\mathrm{Index}\;(\text{\%})=\;\frac{\mathrm{gonad}\;\mathrm{wet}\;\mathrm{weight}}{\mathrm{whole}\;\mathrm{wet}\;\mathrm{weight}}\;\times \;100$$

### (g) Kelp yield and CHNS analysis

**Figure 3.**
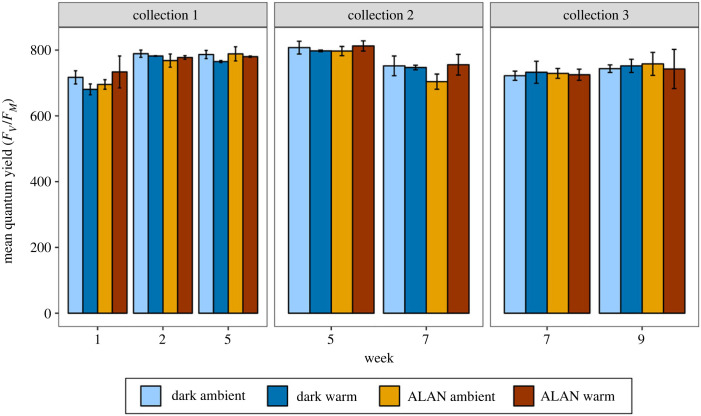
Effect of light (ALAN or dark night) and warming (ambient or warm) on mean quantum yield (*F_V_*/*F_M_*) (±s.e.) of treated *E. radiata* by week. Kelp was collected in weeks 1, 5 and 7, and the graph is faceted by this collection group (collection 1, 2 and 3, respectively). Error bars represent ± s.e.

Although replication of treatments in kelp tanks was low (*n* = 2), we assessed condition of ‘treated kelp’ to examine any obvious drivers of differences, if any, in gonad index and grazing rates of urchins fed ‘treated kelp’. Kelp sampling was conducted alongside urchin sampling in weeks 1, 2, 5, 7 and 9. Since kelp was collected again before weeks 5 and 7, in these weeks, we sampled kelp from both the old and new collection (figure 3). At each timepoint, a 15 cm piece of kelp blade was cut from a randomly selected individual in each tank. The piece of kelp was placed in foil for at least 15 min, before Pulse Amplitude Fluorometry (PAM) was used to measure maximum photosynthetic quantum yield (*F_v_*/*F_m_*) of photosystem II. The 15 cm piece of kelp was then frozen at −80°C for carbon and nitrogen analysis. Whole kelp samples were rinsed in distilled water, dried in an oven at 60°C, and ground in a ball bearing grinder. CHNS analysis was undertaken at Macquarie University using a Vario MICRO cube elemental analyser (Elementar Analysensysteme GmbH, Germany), to measure kelp carbon and nitrogen content and C : N ratio.

### (h) Statistical analysis

To compare grazing rates of *C. rodgersii* across treatments, linear mixed-effect models were fitted using the ‘lmer’ function from the ‘lme4’ package [65]. We ran separate models for urchins fed untreated ‘fresh kelp’ (*n* = 10; *N* = 40), and for urchins fed ‘treated’ kelp (*n* = 4; *N* = 16) (i.e. kelp kept in tanks with the same light and temperature treatments as urchins). Daily grazing rate of urchins (divided by body mass) was the response variable with light (fixed, categorical with two levels: ALAN and dark), temperature (fixed, categorical with two levels: warm and ambient) and week (fixed, numerical with five levels: 1, 2, 5, 7, 9) as interactive factors (three-way interaction), and tank as a random effect. We fitted similar linear models for gonad index using the ‘glm’ function in base R, with light (fixed, categorical with two levels: ALAN and dark) and temperature (fixed, categorical with two levels: warm and ambient) as interactive factors (two-way interaction). Models for gonad index were run without the fixed factor of week or the random effect of tank since gonad index was only measured at the end of the experiment for all urchins.

To compare kelp condition across treatments (*n* = 2; *N* = 8), linear mixed-effects models were fitted using the ‘lmer’ function from the ‘lme4’ package. Photosynthetic yield, log-transformed C : N ratio, total C content (%) and total N content (%) were used as the response variables in separate models with light (fixed, categorical with two levels, ALAN and dark), temperature (fixed, categorical with two levels, warm and ambient) and week (fixed, numerical with five levels, 1, 2, 5, 7, 9) as interactive factors (three-way interaction), and tank as a random effect. C : N ratio was log transformed before analysis as recommended for ecological stoichiometry ratios [66]. Models were run separately for each kelp collection group (i.e. collection 1, 2 and 3) due to the different amount of time each kelp collection group could be maintained.

Gaussian distribution was determined to be the most appropriate distribution for all models and significance was assessed using a likelihood-ratio test via the ‘Anova’ function (type II) from the ‘Car’ package [67]. *Post hoc* contrasts were performed using the R package ‘emmeans’ [68].

## 3. Results

### (a) Kelp grazing rates

**Figure 1.**
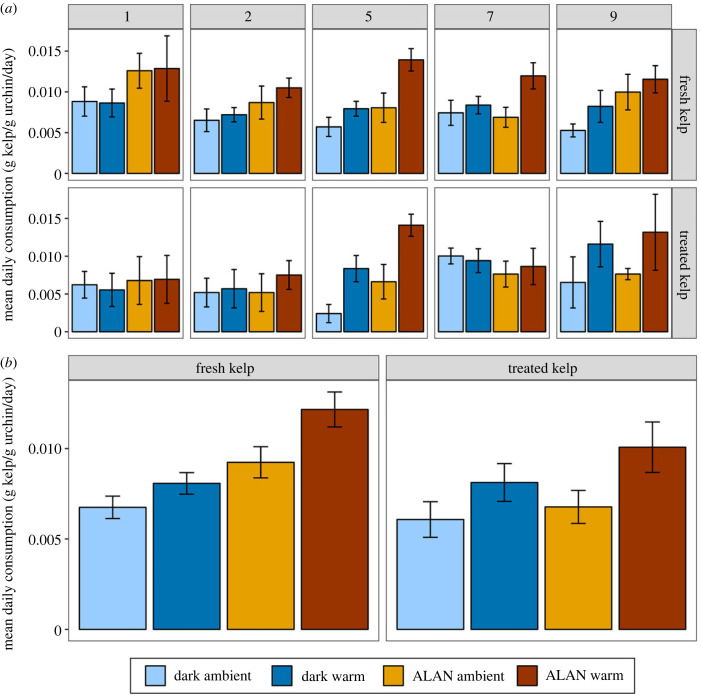
Effect of light (ALAN or dark night), warming (ambient or warm) and kelp treatment (fresh or treated) on mean daily grazing of kelp (±s.e.) by *C. rodgersii* standardized by urchin wet weight. Measurements were repeated on two sample nights for each tank per week. Graph (*a*) shows values by week. In graph (*b*), means and s.e. are averaged across weeks for fresh kelp, as there were no interactive effects of weeks or any week effects on grazing rates for urchins fed fresh kelp. Left graph in (*b*) shows treatment with urchins fed fresh kelp (*n* = 10), right graph in (*b*) shows treatments with urchins fed treated kelp (*n* = 4) (i.e. urchins were fed kelp exposed to the same light and heat treatments). Error bars represent ± s.e.

Across weeks, kelp grazing was significantly higher in ALAN treatments compared to dark treatments, for urchins fed fresh kelp (table 1; figure 1*b*; electronic supplementary material, table S2). Grazing rate was also significantly higher in warm treatments compared to ambient treatments for urchins fed fresh kelp, across weeks (table 1; figure 1*b*; electronic supplementary material, table S2). There was no significant effect of week and no interactive effects on grazing rates for urchins fed fresh kelp (table 1; electronic supplementary material, table S2). For urchins fed treated kelp, grazing rates were higher in warm treatments compared to ambient treatments overall, and grazing increased significantly with time (table 1; figure 1*a*). There was no significant effect of ALAN on grazing rates of urchins fed treated kelp, and no significant interactions between variables (table 1).

**Table 1.** Analysis of grazing rates for *C. rodgersii* fed (*a*) fresh kelp (collected twice a week) and (*b*) treated kelp (kelp exposed to the same temperature and light treatments as the urchins). Grazing was measured as g kelp/day divided by urchin wet weight (g) and averaged over two sample days for each week. A linear mixed model was used with grazing rate as a response and temperature, light (ALAN or dark) and week as interacting factors. Tank was included as a random factor. Significant results at *p* < 0.05 are in italics.

	(*a*) fresh kelp			(*b*) treated kelp		
	χ^2^	d.f.	*p*-value	χ^2^	d.f.	*p*-value
light	9.374	1	*0.002*	0.994	1	0.319
temp	3.908	1	*0.048*	4.060	1	*0.044*
week	1.416	1	0.234	8.096	1	*0.004*
light × temp	0.553	1	0.457	0.223	1	0.636
light × week	0.030	1	0.863	0.043	1	0.835
temp × week	1.630	1	0.202	1.964	1	0.161
light × temp × week	0.002	1	0.968	0.006	1	0.936

### (b) Gonad index

**Figure 2.**
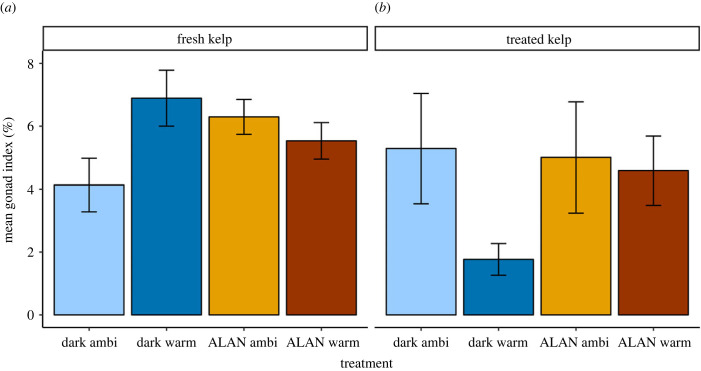
Effect of light (ALAN or dark) and temperature (ambient or warm) on mean gonad index (±s.e.) of urchins fed fresh kelp (*a*) or treated kelp (*b*). Gonad index is gonad wet weight as a proportion of total wet weight (%). Panel (*a*) shows treatments with urchins fed fresh kelp (*n* = 10), (*b*) shows treatments with urchins fed treated kelp (*n* = 4) (i.e. urchins were fed kelp exposed to the same light and heat treatments). Error bars represent ± s.e.

There was a significant interactive effect of ALAN and temperature on gonad index for urchins fed fresh kelp (table 2; figure 2; electronic supplementary material, table S3). Gonad index was significantly higher in dark warm treatments compared to dark ambient treatments (table 2; figure 2; electronic supplementary material, table S3), and in ALAN ambient treatments compared to dark ambient treatments. However, there was no significant difference between ALAN warm and ALAN ambient treatments, or between ALAN warm and dark warm treatments (table 2; figure 2; electronic supplementary material, table S3). Mean gonad index in urchins fed treated kelp (kelp exposed to the same treatments as urchins) was lowest in dark warm treatments, however, differences were not statistically significant (table 2). We found no significant effects of light or temperature on gonad index, and no significant interactions (table 2; figure 2; electronic supplementary material, table S3).

**Table 2.** Analysis of gonad index across light and temperature treatments, for urchins fed (*a*) fresh kelp and (*b*) treated kelp. Significant results at *p* < 0.05 are in italics.

	(*a*) fresh kelp			(*b*) treated kelp		
	χ^2^	d.f.	*p*-value	χ^2^	d.f.	*p*-value
light	0.301	1	0.583	0.839	1	0.360
temp	1.850	1	0.174	2.030	1	0.154
light × temp	5.744	1	*0.017*	1.255	1	0.263
*post hoc*	dark: warm > ambient					
	ALAN: warm = ambient					
	ambient: ALAN > dark					
	warm: ALAN = dark					

### (c) Kelp condition measurements

Kelps were collected at three different times, referred to here as kelp collection groups 1, 2 and 3, respectively. Photosynthetic yield increased significantly over time across treatments in collection group 1 (electronic supplementary material, table S4; figure 3). However, there were no significant effects of light or temperature on yield, and no significant interactions between variables (electronic supplementary material, table S4; figure 3). In collection group 2, there was a significant interactive effect of light and time on photosynthetic yield, and *post hoc* tests showed yield decreased over time in both ALAN and dark treatments (electronic supplementary material, table S4; figure 3). We found no effects of temperature on photosynthetic yield for collection group 2. For collection 3, there were no significant effects of light, temperature or week on photosynthetic yield (electronic supplementary material, table S4; figure 3).

**Figure 4.**
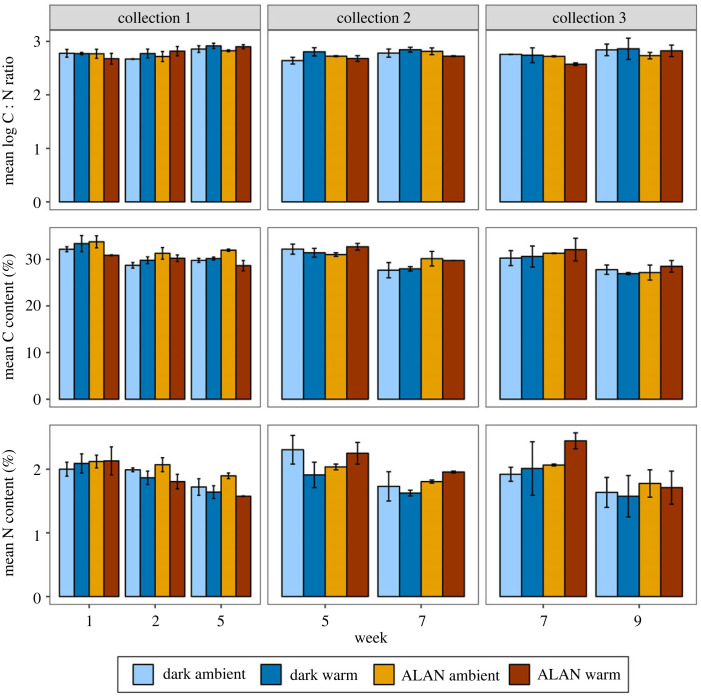
Effect of light (ALAN or dark) and warming (ambient or warm) on mean (±s.e.) (*a*) log-transformed C : N ratio, (*b*) C content and (*c*) N content of treated kelp by week and treatment. Juvenile kelp was collected in weeks 1, 5 and 7, and the graph is faceted by this collection group (collection 1, 2 and 3, respectively). Error bars represent ± s.e.

In all kelp collection groups, C : N ratio significantly increased over time across treatments (electronic supplementary material, table S5; figure 4*a*). In collection 2, there was a significant interaction between light and warming treatments for C : N ratio, however the *post hoc* tests showed no significant pairwise differences (electronic supplementary material, table S5). There were no other effects of light or warming on C : N ratio, and no other significant interactions in any collection groups. Carbon content significantly decreased over time across treatments in all collection groups (electronic supplementary material, table S6; figure 4*b*). For collection 1, carbon content of kelp was significantly higher in ALAN ambient treatments compared to ALAN warm treatments (electronic supplementary material, table S6; figure 4*b*). There were no other effects of light or warming on carbon content, and no other significant interactions. Nitrogen content significantly decreased in all kelp collection groups over time across treatments (electronic supplementary material, table S7; figure 4*c*). In collection 2, there was a significant interactive effect of light and temperature on nitrogen content, however *post hoc* tests showed no significant pairwise differences (electronic supplementary material, table S7; figure 4*c*). There were no other effects of light or warming on nitrogen content, and no other significant interactions.

## 4. Discussion

Ocean warming and ALAN are global stressors that can have important impacts on marine ecosystems. We tested the potential for these stressors, alone and combined, to alter trophic interactions between *C. rodgersii* and *E. radiata*. As separate stressors, ALAN and warming both significantly increased grazing rates of *C. rodgersii* fed fresh kelp, but counter to our predictions, there were no interactive effects of ALAN and warming on grazing rates. There were, however, significant interactions between ALAN and warming on gonad index for urchins fed fresh kelp. Gonad index was significantly higher with warming, but only for dark treatments. Similarly, gonad index was higher when urchins were exposed to ALAN, but only in ambient temperature treatments. This study shows that ALAN and warming may independently increase grazing pressure of *C. rodgersii* in the marine environment.

For urchins fed fresh kelp, grazing rates were higher in warm treatments, likely due to an increase in metabolic rate with warming, as seen in other urchin species (e.g. [34,35]). Similarly, grazing rates of urchins fed ‘treated kelp’ increased in warm treatments compared to ambient temperature treatments. This indicates that effects of warming on urchin grazing are likely to be consistent when both urchins and kelp are exposed to warming simultaneously. Our results show that ocean warming may increase the grazing pressure of *C. rodgersii* on rocky reefs, as suggested by Provost *et al*. [27]. Although mean difference in warming levels between treatments was reasonably low in this study (+1°C), this was enough to cause effects on urchin grazing rates. Predicted ocean warming in south-eastern Australia in the near future, and as observed in recent heatwaves [69], is thus likely to have significant ecological effects on local reefs. South-eastern Australia has been identified as an ocean warming hotspot [70], due to the warming and strengthening poleward flow of the East Australian current [56,71]. In addition, *E. radiata* is highly vulnerable to increased temperatures caused by climate change, which could be exacerbated by increased sea urchin grazing [25–27].

Although we did not find interactive effects of ALAN and warming on grazing rates, urchins exposed to ALAN and fed fresh kelp had higher grazing rates compared to those exposed to normal day/night cycles. This was contrary to our predictions, and contrasts with previous studies that found no impact of ALAN on grazing rates for the urchins *Arbacia lixula* and *Paracentrotus lividus* [36], and a reduction in grazing rate of *Heliocidaris crassispina* with ALAN [37]. However, responses to ALAN can be highly species specific due to differences in biology and ecology, so this difference in response may reflect differences in photosensitivity and behaviour between species. ALAN has been observed to increase consumption rates in other invertebrates [14,43]. The increase in grazing rates of *C. rodgersii* in ALAN treatments observed here might be due to a behavioural shift, whereby feeding was no longer restricted to nocturnal grazing. Alternatively, grazing rates may have increased under ALAN due to an increase in stress, which could, in turn, increase energy demands and metabolic rates of urchins [43]. By contrast, for urchins fed treated kelp, there was no significant effect of ALAN on grazing rates. This may indicate a negative effect of ALAN on kelp palatability that counterbalanced the effect of increased grazing rates in urchins. While there were few differences detected between kelp treatments (see below), there may have been other effects on kelp that impacted grazing, e.g. changes to kelp microbiota [29,30]. While the light intensity used in this study was higher than ALAN levels that currently occur on some shorelines in Sydney Harbour [60], it is still within relevant light levels that can occur directly under infrastructure such as ports and marinas [13]. Additionally, the levels applied here are useful for understanding the mechanisms and future impacts of ALAN, as ALAN is increasing in intensity and extent with growing urbanization and use of LEDs [72]. For urchins fed fresh kelp, there was no significant effect of time on grazing rate, showing treatment effects were consistent over the experimental period (nine weeks). For urchins fed treated kelp, consumption rates increased significantly over time across treatments. This indicates that urchins may have acclimatized to the treated kelp diet over time, possibly due to treated kelp (across treatments) having different microbial or nutritional qualities than wild kelp due to being kept in the aquaria.

We found significant interactive effects of light and warming on gonad index of urchins fed fresh kelp. Gonad index was significantly higher in dark warm treatments compared to dark ambient treatments, for urchins fed fresh kelp. However, the effect of warming on gonad index was not detectable when urchins were exposed to ALAN. Similarly, gonad index was higher in ALAN ambient treatments compared to dark ambient treatments, but there was no effect of ALAN on gonad index in warm treatments. Gonad index was measured at the end of the experiment, so differences in gonad index could have been due to increases or decreases in gonad index over this time. As this experiment was conducted during the *C. rodgersii* spawning period [46], increased gonad growth due to increased consumption rates is unlikely. It is possible that urchin spawning differed between treatments, as *C. rodgersii* spawning is influenced by photoperiod and temperature [46]. However, the actual difference in gonad index between treatments was small, with the largest mean difference between treatments being ±2.76% GI (electronic supplementary material, table S3), and unlikely to be biologically meaningful. Studies conducted during the gonad growth period of *C. rodgersii* (austral spring–summer [46]) could help clarify the effect of artificial light and warming on gonad production.

As the primary objective of this study was to assess the effects of ALAN and warming on the grazing rates of urchins, replication to assess how these stressors affected the kelp themselves was low (*n* = 2). Therefore, these results should be interpreted with caution. We observed no significant differences in photosynthetic yield, C : N ratio or N content between kelp treatments. In collection 1, carbon content was significantly higher for kelp in the ALAN ambient treatment compared to the ALAN warm treatment. However, there was no difference in carbon content between treatments in other collection groups. This contrasts with studies that have found higher C : N ratio, lower nitrogen content and lower photosynthetic rates of *E. radiata* along latitudinal temperature gradients [25,26]. Nevertheless, manipulative experiments testing the effects of temperature on *E. radiata* have similarly found no difference in C : N ratio or photosynthetic yield with warming [29,30,73]. Additionally, kelp was only maintained in treatments for three to five weeks, as *E. radiata* had to be collected multiple times during the experiment, which could also explain the lack of effects observed. Nevertheless, these results provide insights on the potential mechanisms driving the changes in grazing rates and gonad index of urchins found here. Further experiments with greater replication and longer duration would allow us to fully understand effects of ALAN and warming on *E. radiata*. Additionally, future experiments should analyse interactive effects of ALAN and warming on kelp microbial communities, as these have been shown to differ with warming and affect palatability of *E. radiata* to other urchin species [30].

We showed, for the first time, that both warming and ALAN can increase *C. rodgersii* grazing rates, which could increase the top-down effects of this species on kelp forests in south-eastern Australia, as the occurrence and magnitude of these stressors are predicted to increase along coastlines in Australia and worldwide.

## Data Availability

The data and code that support the findings of this study are openly available in Dryad [74]. Additional data are provided in the electronic supplementary material [75].
